# Post-Traumatic Stress Associated with Telework-Related Job Limitation in Latin America

**DOI:** 10.3390/ijerph20136240

**Published:** 2023-06-28

**Authors:** Mariluz Briceño, Grecia Noblejas, Jose Armada, Victor Serna-Alarcón, Martín A. Vilela-Estrada, Víctor Juan Vera-Ponce, Mario J. Valladares-Garrido, Christian R. Mejia

**Affiliations:** 1 Carrera de Medicina Humana, Facultad de Ciencias de la Salud, Universidad Peruana de Ciencias Aplicadas, Lima 15113, Peru; 2 Faculty of Business Sciences, Universidad Continental, Huancayo 12000, Peru; 3 Hospital Regional José Cayetano Heredia, EsSalud, Piura 20002, Peru; 4 Escuela Profesional de Medicina Humana, Universidad Privada Antenor Orrego, Trujillo 13001, Peru; 5 Instituto de Investigación en Ciencias Biomédicas, Universidad Ricardo Palma, Lima 15039, Peru; 6 Facultad de Psicología, Universidad Tecnológica del Perú, Lima 15046, Peru; 7 South American Center for Education and Research in Public Health, Universidad Norbert Wiener, Lima 15046, Peru; 8 Oficina de Epidemiología, Hospital Regional Lambayeque, Chiclayo 14012, Peru; 9 Asociación Médica de Investigación y Servicios en Salud, Lima 15073, Peru

**Keywords:** stress disorders, posttraumatic, occupational health, COVID-19, Latin America

## Abstract

During the pandemic, there has been evidence of work limitations during telework, which are believed to cause mental health problems. Our objective was to assess the association between perceived work limitations during telework and posttraumatic stress during the COVID-19 pandemic in Latin America. A cross-sectional study was conducted in Latin America in 2020. Exposure was measured by self-reporting using a self-perception questionnaire; the SPRINT-E questionnaire was used for outcome measurements. Generalized linear models were applied. Of 1329 participants, 15.2% (n = 202) had posttraumatic stress. In a multivariate analysis, the highest frequency of posttraumatic stress was found among those with moderate depression or more (PR = 1.29; 95% CI: 1.03–1.61), moderate or more anxiety (PR = 2.34; 95% CI: 1.61–3.41), and moderate or more stress (PR = 2.45; 95% CI: 1.46–4.12). In conclusion, there is an association between perceived work limitations during telework and posttraumatic stress in Latin American workers. For this reason, it is recommended that occupational physicians, companies, and institutions assess the frequency of posttraumatic stress and monitor the mental health of workers.

## 1. Introduction

Teleworking is work carried out outside the company’s facilities and in which information and communication technologies are used [1,2]. In this way, the worker could carry out his professional activities, full or part-time, without being physically present in the company [3]. Before the COVID-19 pandemic, teleworking in the European Union ranged from 10 to 30%. In the United States, 20% of teleworkers worked from their residence or other alternative space, 16% in Japan, and only 1.6% in Argentina [4]. In Peru, Law No. 30036 related to teleworking was approved in 2013 [5]. However, until 2018, only some companies opted for this new labor modality [5,6].

The arrival of the pandemic generated by SARS-CoV-2 caused deterioration not only in physical health [7,8,9,10,11,12] but also in mental health [13,14,15]. For this reason, each country’s highest authority proposed a temporary solution, which consisted of forcing the closure of companies and opting for teleworking. A study in Spain showed that 43% of the companies have preserved their productivity, 26% of the companies accepted the total work stoppage, 4% of the companies had a significant increase in work activity, and 1% of the companies opted for definitive closure [16]. In addition, the difficulties of companies carrying out teleworking have been evidenced, such as the need for more hierarchy, by not being able to direct, supervise, and train personnel remotely. Likewise, it was difficult to show instructions on the use of technological tools necessary for the execution of work [17].

Furthermore, obstacles have been encountered due to teleworking, including interruptions during work activities and the overlap between work and personal life, which has generated family conflicts, especially for employers with children. These events have been reported in Europe and the United States [17,18]. These inconveniences during the working day could be associated with more significant stress and less well-being in the working population. As reported by Eurofound and the European Commission, 41% of teleworkers reported high levels of stress, and 42% reported a higher tendency to present sleep disorders [18].

Work limitations related to teleworking may cause mental distress. One of the most severe is posttraumatic stress disorder (PTSD), which affects nearly 4% of the adult population of the United States each year, and it is estimated that 1 in 11 people will develop PTSD in their lifetime [19]. This has been seen in the Ebola virus outbreak in West Africa [20]. In Sierra Leone, up to 76% were found to have PTSD [20]. Post-pandemic H1N1 influenza has also been reported in China [21]. In Peru, it was found in a population affected by floods caused by the El Niño Costero phenomenon [22]. Currently, PTSD has been reported as one of the most frequent diagnoses during the pandemic [23,24,25,26]. Likewise, an increase in depressive symptoms, anxiety [27,28], suicide risk [29], psychological distress [30], and sleep problems [31] were due to the SARS-CoV-2 pandemic [32].

For this reason, a greater frequency of posttraumatic stress has developed during the pandemic. However, the new work modality and its limitations were added, which could increase this condition. Work limitations may generate stress and other mental problems, such as anxiety and depression, affecting work performance. Extended hours, less time for housework, and the overlap between work and family life may cause discouragement, a greater desire to give up work, inadequate interpersonal relationships, and even conflicts within the family [18,33]. A high work overload represents one of the most common factors that cause work stress, because it negatively affects the productivity and efficiency of workers. Moreover, the instability of the financial situation can greatly influence mental health [34]. Technological changes and low wages are also sources of stress due to forced adaptation to a new environment [35]. All these conditions are consistently present and could explain the influence of work limitations related to telework on the development of PTSD. Therefore, the aim of this study was to determine the association between perceived work limitations during teleworking and the frequency of posttraumatic stress in the context of the SARS-CoV-2 pandemic in Latin America in 2020.

## 2. Materials and Methods

### 2.1. Study Design

An analytical cross-sectional study was conducted based on a secondary data analysis from a previous study in the general population [36]. The primary study was conducted between June and August 2020 in 18 Latin American countries, of which 13 countries were selected (Bolivia, Chile, Colombia, Costa Rica, Ecuador, El Salvador, Guatemala, Honduras, Mexico, Panama, Paraguay, Peru, and Venezuela) as the most representative for this study.

### 2.2. Participants

In this study, 1329 workers between the ages of 18 and 78 were enrolled, of whom 55% were women. The inclusion criteria were the general population aged 18 years and who were willing to participate in the study and answered all questions in the data collection instrument. The exclusion criteria were people who had been studying and/or had not been working at the time of the survey, people who had not answered questions related to work limitations during teleworking, and people who had not answered the Short Posttraumatic Stress Disorder Rating Interview (SPRINT-E). Statistical power was calculated for the crossover of the two main variables, in which it was calculated for the difference in the proportions of the minimum and intermediate labor limitations (12.5% versus 16.7%) and of the minimum labor limitation versus the maximum (12.5% versus 19.0%); in both crosses, powers were obtained that exceeded the minimum required value, 85% and 100%, respectively, so the statistical power was adequate for the proposed objective.

### 2.3. Measures

The outcome was the frequency of posttraumatic stress, for which the SPRINT-E questionnaire was used. This instrument was validated in Spanish in 2013. Regarding the reliability of the SPRINT-E, a Cronbach’s alpha value of 0.916 was obtained for the 11 items that evaluate what the individual experienced during the last month. Item 1 evaluates criterion B “Intrusive reexperiencing”; items 2 and 3 evaluate criterion C “Avoidance and numbness”; item 4 evaluates criterion D “Hyperactivity”; items 5 and 7 evaluate depression and healthy behaviors; items 6, 9, and 10 evaluate the loss of the individual’s functionality; and items 8 and 11 assess the individual’s need for support. The items were measured using a Likert scale with a score from 0 (not at all) to 4 (very much). A symptom is intense if 3 or 4 points are obtained. The “3/7 rule” is applied: A person with 3 or more intense symptoms probably has PTSD; if the person has 7 or more symptoms, the possibility of a false positive is minimal [37]. This variable has two categories. On the one hand, people who have experienced 3 or more symptoms are considered to have posttraumatic stress. On the other hand, people who have experienced 2 or fewer symptoms are considered not to have posttraumatic stress.

The main exposure was perceived work limitations during teleworking, for which a perception report was used in which three categories were obtained. First, those who stated that they had a maximum limitation according to the alternative “I do a few things from my work at home and with many limitations” were considered minimally teleworking. Second, those who stated that they had an intermediate limitation according to the alternative “I do a large part of my work from home and with some limitations” were considered partial teleworking. Third, the maximum teleworking was those who stated that they had a minimum limitation according to the alternative: “The company is giving me all the facilities to do all my work from home”.

### 2.4. Data Collection and Procedures

For the data collection of the original study, a virtual survey was carried out in Google Forms. Members of the Latin American Federation of Scientific Societies of Medical Students (FELSOCEM) were invited to collaborate in the study data collection process through social networks and private WhatsApp and Facebook groups. The survey was available from June to August 2020 during the first wave of the COVID-19 pandemic in Peru.

### 2.5. Statistical Analysis

Stata 16.0 (StataCorp LLC, College Station, TX, USA) was used for the data analysis. In the descriptive analysis, categorical variables were reported using absolute and relative frequencies. Regarding the age variable, its nonnormal distribution was determined using the Shapiro–Wilk test, for which the median and interquartile ranges were expressed. Regarding the bivariate analysis, the Kruskal–Wallis test (age) and the chi-square test (for other categorical variables) were used. Then, generalized linear models (GLMs) were used, with the Poisson family, link log, and robust variance. From this, raw/adjusted prevalence ratios, 95% confidence intervals, and *p*-values were obtained. For the decision to enter the variables into the final adjusted model, biostatistical criteria were used (for those variables that had a *p* < 0.05 value in the bivariate analysis of the GLMs), and the collinearity of the variables was evaluated (it was observed that they were not present, since no variable had a value >0.10, considered the cutoff point for collinearity).

### 2.6. Ethical Approval

The original study was approved by the Research Bioethics Committee of the Universidad Peruana Antenor Orrego (UPAO). Additionally, this study was approved by the Ethics Subcommittee of the Faculty of Health Sciences of the Universidad Peruana de Ciencias Aplicadas (UPC). Participants gave informed consent. All data were collected anonymously, and the confidentiality was respected.

## 3. Results

**Figure 1:**
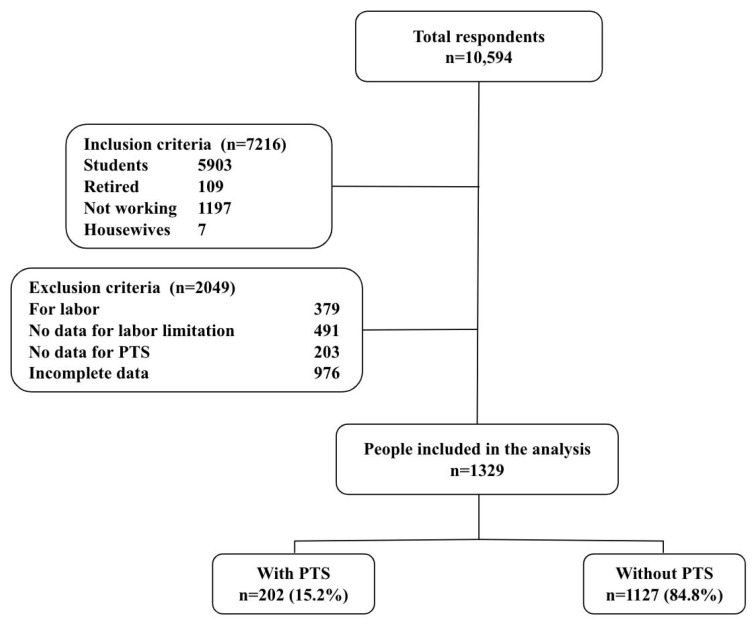
Flowchart of the chosen participants for the research work.

Of 10,594 respondents of the primary study, 7216 were excluded from this analysis, as the participants were students, retirees, nonworkers, and housewives. In addition, 2049 respondents were excluded, because they were unemployed, did not have data for work limitations, did not have data for posttraumatic stress, or had incomplete data (Figure 1).

Regarding the univariate analysis, of the 1329 workers surveyed, 54.7% (*n* = 727) were women; the median age was 38 years (interquartile range: 27–49 years), and the majority had higher education (55.8%) and worked as a teacher (24.0%) (Table 1).

When performing the bivariate analysis, it was found that posttraumatic stress was associated with work limitations (*p* = 0.040), sex (*p* < 0.001), age (*p* < 0.001), history of cardiovascular disease (*p* = 0.003), and respiratory illness (*p* = 0.003), presenting moderate or higher levels of anxiety, depression, and stress (*p* < 0.001 for these three mental pathologies) (Table 2).

In the multivariate analysis, compared to those who had minimal work limitations, 42% more posttraumatic stress was found in those who reported perceived maximum work limitations (PR: 1.42; 95% CI: 1.18–1.69; *p* < 0.001), and 24% more posttraumatic stress was found in those who reported perceiving intermediate work limitations (PR: 1.24; 95% CI: 1.02–1.52; *p* = 0.034). In addition, a higher frequency of posttraumatic stress was found in symptoms of moderate or more depression (PR = 1.29; 95% CI: 1.03–1.61; *p* = 0.025), symptoms of moderate or more anxiety (PR = 2.34; 95% CI: 1.61–3.41; *p* < 0.001), and symptoms of moderate or more stress (PR = 2.45; 95% CI: 1.46–4.12; *p* = 0.001); in contrast, there was a lower frequency of posttraumatic stress in men (PR = 0.64; 95% CI: 0.45–0.92; *p* = 0.016) and in those with a cardiovascular history (PR = 0.35; 95% CI: 0.17–0.73; *p* = 0.005) (Table 3).

Crude and adjusted prevalence ratios (left), 95% confidence intervals (within parentheses), and *p*-values (right) were calculated with generalized linear models (Poisson family, log link function, and adjustments for robust variances and by country of residence).

## 4. Discussion

The study showed an association between posttraumatic stress and work limitations due to telework. This was evidenced by having an intermediate limitation with a weak association. Nevertheless, a maximum limitation had a very strong association (both compared to a minimum limitation). This could be because work limitations may generate stress and other mental problems, such as anxiety and depression, affecting the work performance. Likewise, extended hours, less time for housework, and the overlap between work and family life may cause discouragement, a greater desire to give up work, inadequate interpersonal relationships, and even conflicts within the family [18,33]. A previous study stated that a high work overload represented one of the most common factors that cause work stress, because it negatively affects the productivity and efficiency of workers. Moreover, the instability of the financial situation can greatly influence mental health [34]. In addition, technological changes and low wages are sources of stress due to the forced adaptation to a new environment [35]. All of these conditions are consistently present and could explain the development of PTSD. This association should be studied further, since this finding may potentially serve the generation of occupational surveillance programs.

Other factors influencing the work environment should be considered. A previous study showed that the most frequent fears in a Spanish population related to the SARS-CoV-2 pandemic were the fear that a family member get infected or die from COVID-19, that the virus would continue to spread, and not being able to visit family or friends. However, self-infection or death from COVID-19 was less common. Therefore, these fears may also influence the link between work limitations related to telework and PTSD [38]. Work limitations due to telework must be evaluated by the occupational health area. Additionally, the information provided by health professionals to workers is entirely confidential and aims to improve the quality of life through medical and psychological treatment [33].

Among the other variables that were used for adjustment, men presented a lower association with posttraumatic stress, which was evidenced in other studies, finding that one out of ten women experience posttraumatic stress throughout their lives, higher than men. This may be because women have psychobiological factors that predispose them to affective disorders and because they are more exposed to traumas, such as physical, sexual, and mental aggression, generally at an early age and in their homes [39,40].

In addition, young adults presented a stronger association with PTSD than older adults. This was reinforced by a study showing a significant association between young age and symptoms of depression, anxiety, and stress. This could be explained because older adults often had a better ability to adapt during the COVID-19 pandemic. Furthermore, another study found an annual prevalence of posttraumatic stress in young people between 14 and 34 years of age of 2.9% and between 35 and 65 years of age of 1.3%, while, in adults older than 65 years, the prevalence was 1.1% [23,41].

A history of cardiovascular disease was associated with less PTSD in the workplace. However, other studies have indicated that psychosocial factors, such as emotional factors (severe depression, anxiety, and hostility) and chronic stressors (low social support, unfavorable economic situation, and work stress), are related to cardiovascular diseases that favor the development of atherosclerosis and atherothrombotic complications. In addition, work stress has been considered a new risk factor for developing primarily ischemic cardiomyopathy. Likewise, this is reinforced through two models that describe the link between work stress and coronary disease. The first “job strain” model mentions that workers with a higher workload and low determination have a higher risk of suffering from coronary disease. The second model is the “effort–reward balance”, which indicates that the higher the workload and the less remunerative stimulus, the higher the risk of suffering from coronary disease [42,43,44].

Finally, it was found that suffering from depression, anxiety, or stress was associated with PTSD, which is reflected in other studies reporting that people who have PTSD are more vulnerable to depression, anxiety, or stress. Therefore, it is frequent that, later, they reach the diagnosis of mental disorders. Likewise, there is usually comorbidity between these conditions [41,45].

The main limitation was that the type of study (cross-sectional) does not allow determining the temporality or causality of the relationship between exposure and outcome. Likewise, it is only based on prevalent cases and cannot establish risk factors. However, it does make it possible to establish an association between exposure and disease. In addition, by using a secondary database, the study is limited to those variables measured, since some variables that would have been relevant to this study, such as family income, economic solvency, Internet connection, and technological equipment, were not measured. Likewise, the variable “Perception of presented work limitations” is evaluated subjectively based on a self-report of the perception of each individual on the performance of their work functions. These variables, since they are not validated, will probably not be able to adequately measure the information. However, it is interesting to know the perception that Latin American workers have regarding their work environment.

Finally, the original study could present some selection bias, because it is a non-probabilistic sample (for convenience) and because the data were collected through FELSOCEM students and their acquaintances. Furthermore, it is not certain whether these individuals are a representative sample of the working population of Latin America. Although the results cannot be extrapolated to the general population, it is possible to have a general idea of the behavior of posttraumatic stress in the Latin American population due to the large number of participants.

## 5. Conclusions

There was an association between work limitations related to telework and posttraumatic stress during the COVID-19 pandemic in workers from Latin America. Additionally, an association was found between posttraumatic stress and sex; a history of cardiovascular disease; and anxiety, depression, and stress.

The pandemic has not only significantly impacted the development of PTSD, but work-related adaptations have also contributed to psychological distress. While telework has provided a unique alternative for maintaining work stability, it may not be suitable for everyone, as not all individuals have been adequately prepared for it. To address workers’ mental health issues in a timely manner, it is advised that occupational doctors, companies, and institutions identify employees who are most susceptible to PTSD. This can be achieved through appropriate monitoring and intervention programs, and the Ministry of Labor and Employment Promotion should oversee this process to ensure effective implementation.

## Figures and Tables

**Table 1:** Characteristics of the participants.

Variable	*n*	%
**Gender**		
Female	727	54.7
Male	602	45.3
Age (years) *	38	27–49
**Education**		
High school or less	45	3.4
Baccalaureate	56	4.2
Technical	145	10.9
Superior	741	55.8
Postgraduate	342	25.7
**Profession or occupation**		
Self-employed **	302	23.3
Teaching **	319	24.0
Health	149	11.2
Food **	67	5.1
Economics/administration	69	5.2
Safety **	47	3.5
Mining/construction	42	3.2
**Teleworking by labor constraint**		
Total telecommuting	577	43.4
Regular telework	557	41.9
Minimal telework	195	14.7
**Bereavement/grief for COVID-19**		
Close relative died	72	5.4
Friend died	347	26.1
**Comorbidities**		
Cardiovascular	99	7.5
Respiratory	105	7.9
Obesity	128	9.6
Diabetes Mellitus	45	3.4
**Infected by COVID-19**	24	1.8
**Moderate depression or more**	142	10.7
**Moderate anxiety or more**	237	17.8
**Moderate stress or more**	119	9.0
**Post-traumatic stress**	202	15.2

* Age variable shows medians (interquartile range). ** Some frequencies do not add up to 100%, as they are independent.

**Table 2:** Posttraumatic stress according to work limitations by job and other descriptive characteristics.

Variables	Post-Traumatic Stress		*p* Value
	No *n* (%)	Yes *n* (%)	
**Telework according to labor limitation**			0.040
Total TW/Minimum L	505 (87.5)	72 (12.5)	
Partial TW/Intermediate L	464 (83.3)	93 (16.7)	
Minimum TW/Maximum L	158 (81.0)	37 (19.0)	
**Gender**			<0.001
Female	593 (81.6)	134 (18.4)	
Male	534 (88.7)	68 (11.3)	
Age (years) *	39 (27–49)	32 (25–44)	<0.001
**Education**			0.409
High school or less	38 (84.4)	7 (15.6)	
Baccalaureate	48 (85.7)	8 (14.3)	
Technical	120 (82.8)	25 (17.2)	
Higher	620 (83.7)	121 (16.3)	
Postgraduate	301 (88.0)	41 (12.0)	
**Profession or occupation**			
Self-employed **	256 (84.8)	46 (15.2)	0.951
Teaching **	261 (81.8)	58 (18.2)	0.090
Health	128 (85.9)	21 (14.1)	0.690
Food **	58 (86.6)	9 (13.4)	0.677
Economics/administration	62 (89.9)	7 (10.1)	0.230
Safety **	38 (80.9)	9 (19.2)	0.443
Mining/construction	33 (78.6)	9 (21.4)	0.253
**Bereavement/grief for COVID-19**			
Close relative died	59 (81.9)	13 (18.1)	0.488
Friend died	300 (86.5)	47 (13.5)	0.318
**Comorbidities**			
Cardiovascular	94 (95.0)	5 (5.1)	0.003
Respiratory	81 (77.1)	24 (22.9)	0.023
Obesity	107 (83.6)	21 (16.4)	0.689
Diabetes Mellitus	38 (84.4)	7 (15.6)	0.946
**Infected with COVID-19**	20 (83.3)	4 (16.7)	0.840
**Moderate depression or more**	71 (50.0)	71 (50.0)	<0.001
**Moderate or more anxiety**	136 (57.4)	101 (42.6)	<0.001
**Moderate or more stress**	46 (38.7)	73 (61.3)	<0.001

TW: Telework. L: Labor limitation due to virtuality. * Age variable shows the median (interquartile range). ** The frequencies do not add up to 100%, as they are independent. The *p*-values were obtained with the Kruskal–Wallis statistical test (age) and chi-square (for other variables).

**Table 3:** Bivariate and multivariate analyses of work limitations during telework and posttraumatic stress.

Variable	Bivariate Analysis PR 95% CI *p* Value	Multivariate Analysis PR 95% CI *p* Value
**Labor Limitation**		
Total TW/minimum L	Ref.	Ref.
Partial TW/Intermediate L	1.34 (1.02–1.75) 0.033	1.24 (1.02–1.52) 0.034
Minimum TW/Máximum L	1.52 (1.16–2.00) 0.003	1.42 (1.18–1.69) <0.001
**Gender**		
Female	Ref.	Ref.
Male	0.61 (0.48–0.78) <0.001	0.64 (0.45–0.92) 0.016
Age (years) *	0.98 (0.97–0.99) <0.001	0.99 (0.98–1.00) 0.060
**Education**		
High school or less	Ref.	-
Baccalaureate	0.92 (0.52–1.61) 0.766	-
Technical	1.11 (0.49–2.51) 0.805	-
Higher	1.05 (0.66–1.66) 0.836	-
Postgraduate	0.77 (0.49–1.22) 0.266	-
**Profession or occupation**		
Self-employed	0.99 (0.78–1.26) 0.938	-
Teaching	1.27 (0.94–1.72) 0.117	-
Health	0.92 (0.55–1.53) 0.744	-
Food	0.88 (0.62–1.24) 0.461	-
Economics/administration	0.66 (0.22–1.97) 0.452	-
Security	1.27 (0.76–2.14) 0.366	-
Mining/construction	1.43 (0.97–2.10) 0.068	-
**Bereavement/grief for COVID-19**		
Close relative died	1.20 (0.98–1.47) 0.075	-
Friend died	0.86 (0.73–1.01) 0.058	-
**Comorbidities**		
Cardiovascular	0.32 (0.15–0.68) 0.003	0.35 (0.17–0.73) 0.005
Respiratory	1.57 (1.18–2.09) 0.002	1.09 (0.84–1.42) 0.516
Obesity	1.09 (0.85–1.39) 0.498	-
Diabetes Mellitus	1.02 (0.56–1.87) 0.938	-
**Infected with COVID-19**	1.10 (0.40–3.00) 0.855	-
**Moderate depression or more**	4.53 (3.60–5.70) <0.001	1.29 (1.03–1.61) 0.025
**Moderate or more anxiety**	4.61 (4.10–5.18) <0.001	2.34 (1.61–3.41) <0.001
**Moderate or more stress**	5.75 (4.19–7.91) <0.001	2.45 (1.46–4.12) 0.001

TW: Telework. L: Labor limitation due to virtuality. * Variables expressed quantitatively.

## Data Availability

Not applicable.
